# Determinants affecting the prognosis of decompressive craniectomy for traumatic brain injury

**DOI:** 10.12669/pjms.36.4.2045

**Published:** 2020

**Authors:** Haitao Jiang, Guangshan Hao, Rui Zhang, Qi Pang

**Affiliations:** 1 Haitao Jiang, Department of Neurosurgery, Shandong Provincial Hospital affiliated to Shandong University, 324 Jingwuweiqi Road, Jinan, 250012, China. Department of Neurosurgery, Liaocheng People’s Hospital, 67 Dongchangxi Road, Liaocheng, 252000, China; 2 Guangshan Hao, Department of Neurosurgery, Liaocheng People’s Hospital, 67 Dongchangxi Road, Liaocheng, 252000, China; 3 Rui Zhang, Department of Neurosurgery, Shandong Provincial Hospital affiliated to Shandong University, 324 Jingwuweiqi Road, Jinan, 250012, China; 4 Qi Pang, Department of Neurosurgery, Shandong Provincial Hospital affiliated to Shandong University, 324 Jingwuweiqi Road, Jinan, 250012, China

**Keywords:** Decompressive craniectomy, Traumatic brain injury, Age, D-dimer, Hypernatremia, Post-traumatic hydrocephalus

## Abstract

**Objective:**

This research was designed to investigate the prognostic determinants of patients with traumatic brain injury (TBI) undergoing decompressive craniectomy (DC).

**Methods:**

The present study was a retrospective single center research including a total of 112 patients undergoing DC for TBI in Liaocheng People’s Hospital between January 2017 and December 2018. The results were measured by Extended Glasgow Outcome Sale (GOSE). The prognostic determinants were identified by univariate and binary logistic regression analysis between the deaths and survivors or favorable and unfavorable outcomes.

**Results:**

At the six-month follow-up, the mortality was 45.5% including 37 (33.0%) patients died within 30 days. The independent prognostic factors of 30-day mortality were age (p=0.033), D-dimer level at admission (p=0.032) and postoperative hypernatremia (p=0.014). Seventy five patients survived more than 30 days after DC, among which 27 (36.0%) patients had unfavorable prognosis (GOSE 1-4) and 48 (64.0%) patients presented favorable prognosis (GOSE 5-8). After 30 days from DC, the occurrence of post-traumatic hydrocephalus(PTH) (p= 0.008) was associated with unfavorable prognosis.

**Conclusions:**

Although DC is an effective treatment for TBI patients, the mortality and morbidity risk remain high. A combination of age, D-dimer level at admission and postoperative hypernatremia may be a good prognostic factor for 30-day mortality. Developing an accurate therapy strategy to prevent and control PTH may be beneficial to the 6-month prognosis for TBI patients undergoing DC.

## INTRODUCTION

Traumatic brain injury (TBI) is a devastating neurological disease for its high morbidity and mortality. Traffic accidents and falls are the main factors contributed to TBI.^1^-^3^ China has a pretty bigger population of TBI patients compared to many other countries and the mortality of TBI is about 13/100-000.^2^ Nowadays, the quality and connotation of TBI study in China have been greatly improved, which has been proved by more and more clinical trials.^2^,^4^,^5^

Normal intracranial physiological process can be disturbed following TBI, resulting in refractory intracranial hypertention, decreased cerebral perfusion pressure and disturbed cerebral blood flow.^6^ Malignant intracranial hypertension is the main cause of death in TBI patients.^7^ Therefore, control of the increased intracranial pressure (ICP) is the key treatment of TBI. Decompressive craniectomy (DC), a sufficient method to reduce intracranial hypertention by partial skull removal, has been commonly recognized as a lifesaving treatment for uncontrollable high ICP after TBI.^8^ However, some randomized controlled trials have demonstrated that DC could not only reduce the intracranial hypertension and mortality,^9^,^10^ but also increased the population of survived patients with severe disability or vegetative state.^9^-^11^

Many efforts have been made to explore prognostic factors associated with DC. Several lines of evidence suggest that some factors, such as age, Glasgow Coma Scale (GCS) score, pupil reactivity, midline shift, Helsinki Computed Tomography (CT) score, postoperative Hct, post-traumatic hydrocephalus (PTH), ∆Hct (postoperative Hct minus initial Hct) and delayed cranioplasty, are important predictors.^1^,^3^-^5^ However, it is still controversial about the surgical indications and prognostic factors in clinical practice.

In this research, we retrospectively collected the parameters of 112 consecutive TBI patients undergoing DC in our hospital, a provincial regional medical center in Shandong Province, and analyzed factors associated with the prognosis.

## METHODS

### Participants

This retrospective cross-sectional research was conducted in the 38-bed neurosurgical intensive care unit (ICU) of Liaocheng People’s Hospital, The study was approved by the Institutional Ethics Committee of Shandong Provincial Hospital affiliated to Shandong University, and written informed consent was obtained from all participants. A total of 112 TBI patients (over 16 years old) who received DC treatment from January 2017 to December 2018 were enrolled. Patients with devastating TBI leading to death within 24 hours, severe combined injury, severe coagulation dysfunction, hypoxia (PaO_2_ <70 mmHg), hypotension (systolic blood pressure <90 mmHg) and incomplete clinical information were excluded. The surgical indications were based on the latest literature report and the guidelines for management of TBI (fourth edition) issued by the Brain Trauma Foundation of America.^8^

### Measurements

Clinical variables were reviewed and collected by trained research staff, including basic characteristics (gender, age, trauma information), early clinical conditions (preoperative GCS score, bilateral pupils’ reactivity and size etc.), coagulation markers at admission (fibrin degradation products (FDP), D-dimer level etc.), CT scan findings (subarachnoid hemorrhage, diffuse axonal injury (DAI) etc.), surgical information (type of DC, intraoperative blood loss etc.), and postoperative complications (hypernatremia, PTH etc.). To evaluate the postoperative influencing factors, six aspects were recorded and analyzed, including anemia on a 3-point scale (>90 g/L, 60-90 g/L, or <60 g/L) within 30 days, hypoalbuminemia on a 4-point scale (>30 g/L, 25-30 g/L, 20-25 g/L, or <20 g/L), hypernatremia on a 4-point scale (135-145 mmol/L, 145-155 mmol/L, 155-165 mmol/L, or >165 mmol/L), hypokalemia on a 4-point scale (3.5-5.5 mmol/L, 3.0-3.5 mmol/L, 2.5-3.0 mmol/L, or <2.5 mmol/L), hyperglycaemia on a 3-point scale (<11.1 mmol/L, 11.1-15.0 mmol/L, or >15.0 mmol/L), and hypercreatinine on a 3-point scale (<115 umol/L, 115-178 umol/L, or >178 umol/L). Patients whose records involving at least three values were selected for the study.

### Outcome Assessment

Extended Glasgow Outcome Sale (GOSE) was selected to assess the outcome at 30-day and 6-month follow-up. GOSE, first presented by B Jennett et al, includes eight outcome categories, from death to upper good recovery.^12^ The outcomes were divided into death and survivor or unfavorable prognosis (GOSE 1-4) and favorable prognosis (GOSE 5-8).

### Statistical Analysis

SPSS software (version 22.0, IBM SPSS Statistics) was used to analyze the research data. Descriptive statistics were presented as frequencies (percentages) or mean ± standard deviation. Continuous variables and discrete variables were compared using Student’s *t* tests and Chi-square tests, respectively. The prognostic factors of 30-day mortality and 6-month were assessed by binary logistic regression analysis. When the p value was less than 0.05, the difference was considered to be statistically significant. Receiver operating characteristic (ROC) curve was employed to evaluate risk factors for predicting 30-day mortality. In addition, outcomes were presented as odds ratios (ORs) (ORs >1 indicated a higher risk) with 95% confidence intervals (CI).

## RESULTS

Clinical variables of 112 TBI patients undergoing DC are presented in Table-I. Patients, enrolled in the present study, included 70 (62.5%) males and 42 (37.5%) females. The mean age was 48.11 ± 14.43 years (ranging 16-78 years). Traffic accidents, 61.4% of which were associated with electric bicycles and tricycles, were the major cause of TBI accounting for 62.5% of the injuries, followed by falling injuries representing 16.1% of the injuries. The mean preoperative GCS score was 5.24 ± 1.97. The pupillary examination identified 31 (27.7%) patients with no pupil dilation, 45 (40.2%) with unilateral mydriasis and 36 (32.1%) with bilateral mydriasis. The brain CT, obtained immediately after admission or before surgery, showed subdural hematoma (76.8%), subarachnoid hemorrhage (81.3%), contusion-associated hemorrhage (86.6%), epidural hemorrhage (25.9%), intraventricular hemorrhage (5.4%) and skull fracture (87.5%).

**Table-I:** The Clinical variables of 112 TBI patients undergoing DC*.

Parameter	Value (n=112)*
Mean age in years	48.11 ± 14.43
***Sex***	
male	70(62.5%)
female	42(37.5%)
***Mechanism of head injury***	
road traffic accident	70(62.5%)
associate with electric bicycles and tricycles	43(38.%)
fall accident	18(16.1%)
slip accident	8(7.1%)
injured by heavy object	6(5.4%)
other	10(8.9%)
Mean preoperative GCS score	5.24 ± 1.97
***Pupils abnormality size***	
no pupil dilation	31(27.7%)
unilateral mydriasis	45(40.2%)
bilateral mydriasis	36(32.1%)
***Pupil reactivity to the light***	
both reacting	32(28.6%)
unilateral reacting	22(19.6%)
bilateralnonreacting	58(51.8%)
***CT scanning findings***	
epidural hemorrhage	29(25.9%)
subdural hematoma	86(76.8%)
subarachnoid hemorrhage	91(81.3%)
contusion-associated hemorrhage	97(86.6%)
intraventricular hemorrhage	6(5.4%)
DAI	9(8.0%)
skull fracture	98(87.5%)
***status of basal cisterns***	
compressed	70(62.5%)
absent	42(37.5%)
midline shift in mm	9.64 ± 4.41
***Coagulation markers at admission***	
FDP (ug/ml)	123.54 ± 51.87
D-dimer level (ug/ml)	56.94 ± 24.80
***Type of DC***	
primary	84(75.0%)
secondary	28(25.0%)
***Decompressive procedure***	
unilatalhemicraniectomy	96(85.7%)
bilatalhemicraniectomy	13(11.6%)
bifrontalcraniectomy	3(2.7%)
***Mortality***	
30-day	37(33.0%)
6-month	51(45.5%)
***Final outcome***	
favorable	48(42.9%)
unfavorable	64(57.1%)

* Values are the number of patients (%) unless noted otherwise. Mean values are presented as the mean ± SD.

The mean distance of midline shift was 9.64 ± 4.41 mm (ranging 0-23.4 mm). Primary DC with hematoma evacuation was performed in 84 (75.0%) patients, while 28(25.0%) patients underwent secondary DC because of uncontrollable ICP. Unilateral frontotemporoparietal hemicraniectomy was performed in 96 (85.7%) cases, bilateral hemicraniectomy in 13 (11.6%) cases and bifrontal craniectomy in 3 (2.7%) cases. The mortality at 6-month follow-up was 45.5%, while 37 (33.0%) patients died within 30 days. The outcome was unfavorable in 64 (57.1%) patients and favorable in 48 (42.9%) patients at 6-month follow-up.

The risk factors predicting 30-day mortality by univariate and binary logistic regression analysis is shown in Table-II. Univariate analysis showed the following predictive parameters with clinically significant: age, preoperative GCS score, bilateral pupils abnormality size and reactivity, type of DC, timing of DC, timing between injury and DC, timing between admission and DC, FDP at admission, D-dimer level at admission, subarachnoid hemorrhage, DAI, status of the temporal horn at the midline shift side before craniotomy, status of basal cisterns, intraoperative blood loss, ∆Hct, postoperative GCS score and platelet level at the first day, postoperative hypernatremia, hypokalemia and hypercreatinine. Binary logistic regressions analysis model was used to eliminate the aliasing effect getting the independent prognostic factors: age (OR 1.139 [95% CI 1.011-1.283]; p=0.033), D-dimer level at admission (OR 1.366 [95% CI 1.027-1.816]; p=0.032) and postoperative hypernatremia (OR 16.931 [95% CI 1.772-161.822]; p=0.014).

**Table-II:** Univariate and binary logistic regression analysis of factors predicting 30-day mortality.

Parameter	Univariate		Multivariate	

	OR (95% CI)	p value	OR (95% CI)	p value
Age	1.072(1.035–1.110)	<0.001	1.139(1.011–1.283)	0.033
Preoperative GCS score	0.624(0.458-0.851)	0.001	2.337(0.803-6.804)	0.120
Bilateral pupils abnormality size	2.269(1.296-3.972)	0.011	3.863(0.306-48.829)	0.296
Bilateral pupils reactivity	2.104(1.247-3.552)	0.012	0.748(0.055-10.211)	0.828
Type of DC	0.353(0.122-1.022)	0.049	0.158(0.003-8.208)	0.360
Timing of DC	0.147(0.032-0.666)	0.005	0.000(0.000-3.704)	0.088
Timing between injury and DC	0.999(0.999-1.000)	0.002	1.009(0.999-1.010)	0.074
Timing between admission and DC	0.999(0.999-1.000)	0.015	0.992(0.982-1.001)	0.077
FDP at admission	1.016(1.006-1.025)	<0.001	0.894(0.795-1.005)	0.061
D-dimerlevel at admission	1.037(1.016-1.058)	<0.001	1.366(1.027-1.816)	0.032
Subarachnoid hemorrhage	5.938(1.303-27.066)	0.011	30.969(0.049-19755.739)	0.297
DAI	8.517(1.672-43.378)	0.009	21.371(0.117-3918.095)	0.249
Status of the temporal hornat the midline shift sidebefore craniotomy	5.333(1.488-19.111)	0.005	16.972(0.570-505.453)	0.102
Status of basal cisterns	13.556(5.246-35.030)	<0.001	18.296(0.984-340.370)	0.051
Intraoperative blood loss	1.001(1.000-1.001)	0.045	1.001(0.999-1.003)	0.496
∆Hct	1.148(1.051-1.254)	0.003	1.431(0.967-2.119)	0.073
Postoperative GCS score at first day	0.372(0.235-0.590)	<0.001	0.706(0.296-1.683)	0.432
Postoperative platelet at first day	0.989(0.979-0.998)	0.046	1.000(0.967-1.034)	0.984
Postoperative hypernatremia	4.137(2.352-7.277)	<0.001	16.931(1.772-161.822)	0.014
Postoperative hypokalemia	1.306(0.874-1.953)	0.024	0.812(0.199-3.311)	0.771
Postoperative hypercreatinine	5.944(2.421-14.598)	<0.001	5.593(0.755-41.432)	0.092

**Fig.1:**
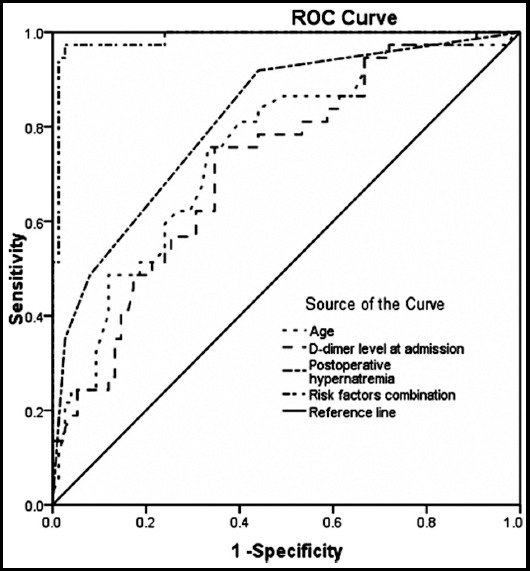
ROC curves of age, D-dimer level at admission, postoperative hypernatremia and risk factors combination for predicting 30-day mortality.

Independent risk factors affecting 30-day mortality were quantitatively analyzed using the ROC curves. When calculated separately, the accuracy of age, D-dimer level at admission and postoperative hypernatremia were 74.1% with a cutoff value of 49.5 years, 67.0% with a cutoff value of 67.4 ug/ml, and 77.7%, respectively. After being adjusted to predict patients of 30-day mortality, the combination of age, D-dimer level at admission and postoperative hypernatremia demonstrated the highest specificity (97.3%) and sensitivity (97.3%). When it was applied to predict prognosis, the best accuracy was 96.4% (Table-III and Fig.1).

**Table-III:** ROC analysis of age, D-dimer level at admission, postoperative hypernatremia.

Parameter	Area under the curve (95% CI)	Youden index	Sensitivity (%)	Specificity (%)	Accuracy (%)
Age	0.751(0.656-0.846)	0.424	75.7	66.7	74.1
D-dimerlevel	0.721(0.622-0.819)	0.410	75.7	65.3	67.0
hypernatremia	0.817(0.734-0.900)	0.492	91.9	56.0	77.7
Combination	0.987(0.969-1.000)	0.906	97.3	97.3	96.4

At the six-month follow-up, the final outcome was favorable in 48 (64.0%) patients among 75 patients who alive 30 days after DC. Risk factors for predicting 6-month prognosis, obtained by univariate and binary logistic regression analysis are depicted in Table-IV. Univariate analysis observed the following indicators with clinically significant: preoperative GCS score, bilateral pupils abnormality size and reactivity, status of basal cisterns, intraoperative blood loss, postoperative GCS score at the first day, postoperative delayed contusion and hematoma, postoperative delayed contralateral hematoma, tracheostomy, hospital acquired pneumonia, PTH, postoperative hypernatremia and hyperglycaemia. PTH (OR 672.702[95%CI 5.561-81370.584]; p= 0.008) was found to be the independent prognostic factor following the binary logistic regressions analysis.

**Table-IV:** Univariate and binary logistic regression analysis of factors predicting 6-month prognosis.

Parameter	Univariate		Multivariate	

	OR (95% CI)	p value	OR (95% CI)	p value
Preoperative GCS score	0.794(0.609-1.036)	0.044	1.283(0.403-4.086)	0.673
Bilateral pupils abnormality size	2.286(1.172-4.457)	0.024	5.551(0.175-175.908)	0.331
Bilateral pupils reactivity	2.253(1.254-4.076)	0.015	1.565(0.136-17.984)	0.719
Status of basal cisterns	10.313(2.547-41.747)	0.001	0.988(0.022-45.293)	0.995
Intraoperative blood loss	1.001(1.000-1.002)	0.030	1.000(0.998-1.003)	0.806
Postoperative GCS score at first day	0.621(0.437-0.881)	<0.001	1.022(0.280-3.727)	0.974
Delayed contusion and hematoma	5.685(2.240-14.425)	<0.001	15.430(0.304-783.702)	0.172
Delayed contralateral hematoma	13.429(1.520-118.629)	0.014	0.279(0.000-215.557)	0.707
Tracheostomy	33.429(4.188-266.807)	<0.001	31.149(0.221-4391.155)	0.173
Hospital acquired pneumonia	7.393(2.215-24.674)	<0.001	24.283(0.255-2316.822)	0.170
PTH	22.000(6.001-80.660)	<0.001	672.702(5.561-81370.584)	0.008
Postoperative hypernatremia	3.006(1.408-6.674)	0.016	8.282(0.690-99.480)	0.096
Postoperative hyperglycaemia	2.690(1.317-5.492)	0.016	1.507(0.198-11.445)	0.692

## DISCUSSION

TBI, as a familiar devastating neurological disease, is an important societal issue imposing huge burden on society and families. Consistent with the previous studies,^1^,^2^,^3^,^5^ the majority of TBI patients in the present study were male adults who were often involved in more adventurous activities. In the present data, the leading cause of TBI was traffic accident representing 62.5% of the injury types including 43 (38.4%) cases relevant to electric bicycles and tricycles. Despite the progress in prevention and treatment, patients do still suffer from undesirable prognosis.^1^,^2^,^5^ In our research, the final outcome was unfavorable in 64 (57.1%) patients, and the 6-month mortality was 45.5%.

### Risk Factors for Short-term Mortality

It is widely accepted that the prognosis of TBI patients is related to age.^13^ For TBI patients requiring DC, age has been widely accepted as one of the most important prognostic factors.^3^,^5^,^14^ Similarly, our findings also indicated that young patients had lower rate of short-term mortality than old counterparts. Meanwhile, we found that the accuracy of 30-day mortality for age was 74.1% with a cutoff value of 49.5 years. Therefore, there is no significant benefit of performing DC for TBI patients after a certain age.

The level of D-dimer is significantly elevated in the early stage of TBI, which is a hot spot in current research. Recent studies have showed that elevated D-dimer level, which intimates the existence of hyperfibrinolysis, is a significant predictor of unfavorable outcomes in TBI patients.^15^-^17^ However, the mechanism of abnormal D-dimer level in TBI patients receiving DC is not well-elucidated. In the study, we found that higher D-dimer level at admission was a significant independent determinant of 30-day mortality, yet no apparent correlation was discovered between D-dimer level and long-term prognosis.

Persistent hypernatremia may be detrimental to the cerebral edema, especially in patients with disrupted blood-brain barrier. In the past two decades, several studies have also evaluated and demonstrated the effect of hypernatremia on mortality after severe TBI.^18^-^20^ In our research, postoperative hypernatremia was presented in 59.8% of patients, most of which suffered mild and severe hypernatremia. Postoperative hypernatremia was an independent risk factor related to short-term mortality, while severe hypernatremia (>165 mmol/L) contributed to a higher mortality. Therefore, postoperative hypernatremia may predict the higher short-term mortality rate and hypernatremia maybe a potential therapeutic target.

### Risk Factors for Long-term Survival

In our research, we comprehensively evaluated preoperative, intraoperative, and postoperative factors in TBI patients underwent DC using binary logistic regression analysis, and found that the independent prognostic factor at the six-month follow-up was PTH (p= 0.008).

PTH, a familiar complication for TBI patients undergoing DC can lead to unfavorable prognosis.^1^,^21^,^22^ Generally, PTH occurs in the first week after DC and then gradually increases over the next three weeks. In our research, the overall incidence of PTH was 19.6%, only 4 (18.2%) of which achieved favorable prognosis. The most possible reason is that delayed diagnosis and therapy of the disease is detrimental to neurological improvement during recovery. PTH was independently associated with unfavorable prognosis, while the presence of subdural hygroma was not associated with favorable prognosis. Therefore, timely and appropriate treatment of PTH should be an important part of postoperative management.

### Limitations of the study

There are several potential limitations in this study. It was a retrospective cross-sectional review of the data, hence the data was not as complete and accurate as planned research. From the statistical perspective, the number of patients was still insufficient to represent the condition of all patients. Finally, the research results merely reflected the experience of a single provincial regional medical center in Shandong Province.

## CONCLUSIONS

Although DC is an effective treatment for TBI patients, the mortality and morbidity risk remains high. A combination of age, D-dimer level at admission and postoperative hypernatremia may be a good prognostic factor for 30-day mortality. Developing an accurate therapy strategy to prevent and control PTH may be beneficial to the 6-month prognosis for TBI patients undergoing DC.

### Authors’ Contributions

**HJ**, **QP:** Designed this study and prepared this manuscript, and are responsible and accountable for the accuracy or integrity of the work.

**RZ:** Collected and analyzed clinical data.

**GH:** Significantly revised this manuscript.
